# Singular Case of Abdominal Disease Simulating Hydr-Ovarium; with the Appearances on Dissection

**Published:** 1816-07

**Authors:** 

**Affiliations:** one of the Editors


### PART I.

### ORIGINAL COMMUNICATIONS.

### *Singular Case of Abdominal Disease simulating Hydr-Ovarium; with the Appearances on Dissection.*

By Mr. JOHNSON, one of the Editors.

ON the 6th of May, 1816, I was called to Mrs. B. a sailor's wife, 40 years of age, who had been landed from a small ship of war, about a fortnight previously, in a very bad state of health. She was now in the eighth month of pregnancy, and complained of a violent and incessant pain in the right side of the abdomen, extending from the umbilicus round to the lumbar vertebræ, and from the floating ribs to the groin. Her pulse was quick, and rather hard; skin hot; bowels confined; urine scanty and high coloured; tongue loaded; thirst; quite sleepless at night.

On examining the abdomen, it appeared unusually large; but, what was more singular, a distinct *sulcus* was felt, running from the scrobiculus cordis to the pubis, on each side of which line the abdominal tumor rounded out, as though there were two impregnated wombs instead of one. Both sides, too, presented nearly the same kind of surface, except that the right felt rather more uniform and elastic than the left. Pressure on every part of the right side gave great pain quite round to the spine.

But rather an imperfect history of the complaint could be obtained, since the origin of the disease appeared to have been laid at an uncertain and probably remote period. She stated, that, for two years at least, she had felt more or less of this pain; but that it was only since the period of quickening, in the present pregnancy, that it became so very distressing. During the last three months, it had been unremitting, and progressively increasing in violence.

On inquiring whether she had any hardness or swelling on that side of the abdomen previous to pregnancy, she answered in the negative; but she said, that, during the last four or five months of a preceding pregnancy, she felt more than usual pain of this side, which subsided considerably after delivery. When questioned respecting the state of the bowels and urinary organs, she replied, that she had always been very regular as to the alvine secretions; but with respect to her water, she was quite the reverse, for, during the last two years, it was always either scanty and high-coloured, or more plentiful and *white like milk.* That when it was in the *latter* state, she was pretty easy; but that when it got scanty, she was in great pain. During the last three months it was scanty. Upon asking her if she could trace her present complaint to any cause, she remarked, that she attributed her ailments to a practice, which, during the suckling of a former child, she had been forced to adopt, in consequence of being at sea with her husband; namely, that of drying all her child's linen and napkins, by wringing them out as hard as possible, and then placing them on the abdomen, next the skin!

From the 6th till the 10th of May, she took aperient medicines; had blisters applied to the side affected; was once bled (the blood exhibiting an inflammatory buff); took diuretics, with nitre and digitalis; had warm fomentations and embrocations externally, together with anodynes internally: all with temporary, and very temporary relief.

On the 10th, she was evidently in labour; and each parturient pain rendered that in the side excruciating.

At four o'clock, on the morning of the 11th, she was delivered of a living child. The placenta was retained for some hours, but ultimately came away. From the moment of delivery, she expressed herself perfectly free from pain. On examining the patient this day, I was surprised to find, that the right side of the abdomen was little diminished in size; but, on the contrary, from the shrinking of the left side, a large tumor appeared, projecting at least four inches above the general level of the belly, and seven or eight inches in diameter. It was turgid, and perfectly even all over its surface; on tapping it gently, a fluctuation was evident, and pressure on it occasioned pain.

No lochia whatever succeeded delivery: but the truce with her old enemy was of short duration. The pain returned, though not in so violent a degree as previous to parturition; while the unhappy woman was attacked with irritative fever, constant micturition, and diarrhœa. For these, such medicines, as were deemed necessary in her state, were prescribed, but without affording any relief to the original complaint, from which she only experienced a temporary immunity by the exhibition of opium.

At this time, she was seen by many medical gentlemen; among others, Dr. Lara, Dr. Grey, Dr. Nagle, Dr. Tobin, Dr. Seeds, &c.; and a general opinion prevailed, that the disease was Ovarian Dropsy. While we were endeavouring to reinstate, as far as possible, the general health of the patient, and while the propriety of puncturing the tumor was maintained by some and denied by others, an event took place, in the night of the 19th of May, which settled the question respecting an operation.

Early in the morning of the 20th of May, the nurse was surprised to find that the patient's bed had been completely drenched, during the preceding night, by a milky fluid, which had spread itself over the floor of the room, and descended, in considerable quantity, into a chamber underneath.—From what source the fluid issued the nurse could not tell, and the patient had that night slept so well, that the passage was involuntary; at least, she was unconscious of the circumstance. She asserted, however, that it was the same as milky urine which she used formerly to make occasionally. It certainly had very little of the urinous smell, and I did not feel disposed to taste it. On pulling down the bed-clothes, the tumor was gone! Manual examination, however, discovered some fulness and fluctuation on that side, though the rise was not very observable above the general level of the abdomen. The pain was also abated, and whitish urine continued to be made in considerable quantities. Notwithstanding this sudden change, the fever, the rapid emaciation, the entire prostration of strength, the sinking countenance, the cold sweats, and colliquative diarrhœa, proclaimed themselves the harbingers of death. Nature, however, struggled four days with these symptoms, during which time a great deal of the same white water was passed involuntarily, as were also the stools. Early in the morning of the 25th of May she expired, the abdomen appearing as flaccid and reduced as though no previous tumor had existed.

The unexpected turn which the disease had latterly taken, began to unhinge the opinions of those who had seen it shortly after parturition; and various were the ingenious and sapient explanations which were given of the sudden discharge of water from the ovarium. It passed along the Fallopian tube; burst into the vagina; into the bladder; nay, after the marvellous case of a woman was read in Mr. Beddingfield's late work, the bladder itself was suspected of being the original seat of the collection. Previous to the discharge of the fluid, one physician hinted an opinion, that the tumor of the abdomen was an abscess of the liver or kidney; but the quality of the fluid evacuated during the subsidence of the tumor gave the ovarian party a decided triumph.

In the midst of this chaos of opinions, I was determined, if possible, to cut the Gordian knot with the scalpel. The result offered little for self-gratulation to the *wisest* or the *proudest* of us; and the same will probably happen to the reader himself.

No trifling address was required to persuade the class of people among whom this poor woman resided, to allow the body to be opened; so nearly invincible are the prejudices against dissection in the lower orders of society. It was not till after many asseverations, that the dead feel no pain, and that *possibly*, thousand of lives might be saved by the investigation of this mysterious tumor, that permission was obtained—and that under the most galling restrictions; particularly that the body should not be cut up like that of a beast; that the parts of generation should not be exposed or touched; that no particle of the corpse should be taken away; that no young pupils should have admission; and finally, that two or three of the gossiping train should be present, in order to see that all the stipulations were observed.

### Sectio Cadaveris—25th of May 1816, Eight Hours after Death.

*(Present, Thomas Seeds, M.D. and myself, with one Assistant.)*

An incision being made from the sternum to the umbilicus, and from the umbilicus to the spine of each ileum, the triangular flap of integuments was everted over the pubis, when the CAPUT COLI first presented itself, much distended, and *apparently* with an immense pouch detached from it and the ascending portion of the colon, which pouch filled up the hollow of the ileum; from the liver to near the groin; and from beneath the anterior parietes of the abdomen back to the spine. On looking down into the cavity of the pelvis, when cleared of the small intestines, the bladder was seen moderately distended; the uterus sufficiently contracted for the length of time after parturition; the ovaria and Fallopian tubes on each side, perfectly distinct, natural, and healthy!

Returning to the colon, on a close examination, and before attempting to remove the parts *ex situ,* I thought I

could perceive something like a sulcus between it and the pouch; and, by a careful and delicate dissection, I separated entirely the caput, and ascending portion of the colon from the bag to which they were so intimately attached, as to appear identified with it. The cyst was next to be detached from the concave surface of the liver; from the hollow of the ileum, and from the posterior part of the peritoneal lining of all the right side of the abdomen round as far as the spine. To these various parts it was most firmly agglutinated, but without a single trace of *recent* inflammation.

In raising the lower portion of the bag from the hollow of the ileum, a large vessel was exposed, lying in the direction of the psoas muscle, which, at first sight, I took for the external iliac artery; but on clearing it a little from the surrounding cellular substance, I was soon undeceived by coming to the iliac artery with its accompanying vein, and also by observing that the vessel itself was tortuous, and unequal in calibre. In some places it was quite as large as the iliac artery, in others smaller. I first carefully traced it upwards, for a considerable way, and ultimately found it enter the body of the sac:—I next traced it downwards, into the cavity of the pelvis; under the Fallopian tube; and into the bladder! Till this moment, the nature of the disease was doubtful; it was now evident, that the vessel so traced was the ureter, while the immense bag, from which it descended, was the capsule of the kidney distended to a hitherto unexampled magnitude!

The ureter, which was very turgid, could be emptied of its contents by pressing the fluid downwards into the bladder, but immediately filled again from the sac. A ligature was passed round it near its entrance into the capsule, and the tube being divided below that, there gushed out nearly two ounces of the same kind of whitish liquid which passed latterly *per urethram.* We now proceeded to detach the capsule from its various attachments to the liver, small intestines, psoas muscle, &c. &c. but found this so tedious and difficult an operation, that the patience of the female spectators became exhausted, and we were forced to hasten the investigation as much as possible, by examining the interior of the bag *in situ.* I had so far separated the capsule, however, that I could pass my hand in all directions round it, except where it adhered to the concave surface of the liver, and where the emulgent vessels entered the bag. I was particularly careful to examine whether the kidney or any part of it remained uninclosed in the cyst; but there was no vestige of the kind. On making an incision into the cyst, six or seven inches in length, we found in its cavity about three pints of the same fluid as came from the ureter, which being sponged out, we had an opportunity of viewing the internal surface of the bag. Its parietes varied a great deal in thickness; from that of a penny piece to that of a shilling. The whole internal surface, however, was highly vascular, and thickly studded with a kind of mammillary, or papillary bodies, varying in size from that of a pin's head, or less, to that of a very small pea. Several thin laminæ, or semicircular septa projected from different parts of the walls of the cyst; but the largest of them never went half across its diameter, so that the freest communication existed throughout the whole cavity; in fact, it was but one cyst, for a large sponge went freely to the bottom of it, and cleared it entirely of its contents.

The bladder was now punctured at the fundus, and nearly a pint of the same kind of fluid as the cyst and ureter contained, flowed out into the pelvic cavity.

It is difficult to calculate the quantity which this cyst may have contained when in its maximum of fulness, immediately after parturition; but from the flaccid state in which it now appeared; from the immense flow of water which took place during its subsidence; and from its great extent and projection externally, before the discharge, we are probably under-rating the quantum much, when we say, that at one time it held five or six quarts of fluid.

What shall we call the disease? It is not a hydatid of the kidney, for there was nothing apparently of the glandular part of the kidney left, unless it was expanded over the internal surface of the capsule.

" Sometimes," says Dr. Bailie, " the true hydatid is formed in the kidnies, having exactly the same nature with that which grows in the liver. It has occurred to me to be able to examine particularly, a case of this kind after death; and I shall describe at some length, what came under my observation. The right kidney in a soldier, was converted into a bag capable of containing at least three pints of fluid, and only a very small part of the kidney at the lower end, retained its natural structure. The bag was of considerable thickness; was obscurely laminated, and had a *cartilaginous hardness upon its inner surface. It was full of hydatids,* which differed very much from each other in their size, some of them being as large as a small orange, and others not larger than the head of a pin. The fluid in many was transparent, but in some hydatids, it resembled whey."—*Morbid Anatomy.* P. 279.

It is evident, that this was quite a different disease from that described by Dr. B. The inner membrane, in the present case, was highly vascular, and, without doubt, a secreting surface; while nothing like hydatids was to be seen. It appears in fact, that, from some cause or other, the pelvis of the kidney had become distended to the size of the interior surface of the present bag, while the glandular substance had either become entirely absorbed, or was expanded between the pelvic and capsular tissues, forming probably the papillary bodies above described, and still retaining its secretory function.

I have searched Morgagni in vain, for any thing "*simile aut secundum*" to the present case, except the following passage, which strongly supports the abovementioned opinion of the nature and origin of the enlargement. "In the *Acta Eruditorum Lipsientia*, an observation is extant, made by Groenvelt on a calculous girl, whose *ureters* resembled one of the *small intestines*, by their capacity being enlarged. And Mauchartus saw the same canals, in an old man afflicted with strangury, *inflated like the intestinum ileum, from urine like butter milk;* at the same time that the kidnies were very large and unequal, and had their *pelves distended to the magnitude of an egg*."—De Causis et Sedibus. Epist. 40. Art. 24.

The previous history and *post mortem* appearances of the disease, leave no doubt that it had been advancing for at least two years, if not more. Throughout the whole circle of its attachments, not a trace of recent inflammation was to be seen; every part appearing in colour and texture, as though it had been an original formation. Yet, the sudden increase, and progressive aggravation of pain, after the period of quickening, during the last pregnancy, evince that the size of the cyst, or, in other words, the distention of the kidney and ureter, was rapidly augmented in the three last months of utero-gestation; and *that*, probably, by the pressure of the uterus on the ureter impeding the passage of water to the bladder, while the same pressure on the cyst occasioned the pain.

Altogether, the case, I should venture to surmise, stands *unique* in the morbid anatomy of the urinary organs. The pelvis and capsule of a kidney distended so as to contain at least five or six quarts of water, and separated only by a thin and doubtful partition of the original glandular substance, will not often be presented to the eye of the medical philosopher, and cannot be deemed unworthy of record.

May the disease be termed "HYDRO-RENAL DISTENTION?" Whether or not the imprudent practice of applying damp linen to the parietes of the abdomen, had here any share in the production of the disease, can be a matter of conjecture only; as must also the *manner* in which it produced that effect. The occasional appearance of *whitish urine,* however, from the commencement of the disorder, and the invariable exacerbations of pain in the lumbar region when the water became scanty or assumed its natural or a higher colour, ought not to be forgotten, as they may furnish pathological hints on a future occasion. Whitish urine is one of the most frequent symptoms in many complaints of the urinary organs, particularly stricture, dysuria, calculus, &c. and very probably arises from *hydro-renal distention,* though it is often attributed to affection of the bladder itself. As the disease, in this instance, simulated hydro-ovarium, and was very generally taken for such; may not the same have sometimes happened before, and passed unquestioned for want of *post mortem* examination?

The operation would have proved fatal, had the patient's health been in ever so good a condition; and for this reason, that where the tumour was most prominent, (about six inches from the umbilicus) and where of course the puncture would have been made, no attachment between the cyst and peritonæum existed, and consequently when the trocar was removed, or the volume of the cyst much reduced, the water would have flowed among the abdominal viscera! This may teach us caution in opening abdominal tumors. Other circumstances in the health being favourable, there could not possibly be seen a tumor which presented fairer inducements for an operation than this. It was prominent; circumscribed; immediately beneath the integuments. The fluctuation unequivocal. Every thing tempting to, and *apparently* sanctioning, an operation; yet that operation would *in itself* have been almost certainly fatal.

J. JOHNSON.

St. George's Square, Portsea,
June 1, 1816.

